# Epitope-Specific Suppression of IgG Responses by Passively Administered Specific IgG: Evidence of Epitope Masking

**DOI:** 10.3389/fimmu.2017.00238

**Published:** 2017-03-06

**Authors:** Joakim J. E. Bergström, Hui Xu, Birgitta Heyman

**Affiliations:** ^1^Department of Medical Biochemistry and Microbiology, Uppsala University, Uppsala, Sweden

**Keywords:** FcγR, complement, sheep erythrocytes, IgG-mediated immune suppression, rhesus prophylaxis, rhesus D antigen

## Abstract

Specific IgG, passively administered together with particulate antigen, can completely prevent induction of antibody responses to this antigen. The ability of IgG to suppress antibody responses to sheep red blood cells (SRBCs) is intact in mice lacking FcγRs, complement factor 1q, C3, or complement receptors 1 and 2, suggesting that Fc-dependent effector functions are not involved. Two of the most widely discussed explanations for the suppressive effect are increased clearance of IgG–antigen complexes and/or that IgG “hides” the antigen from recognition by specific B cells, so-called epitope masking. The majority of data on how IgG induces suppression was obtained through studies of the effects on IgM-secreting single spleen cells during the first week after immunization. Here, we show that IgG also suppresses antigen-specific extrafollicular antibody-secreting cells, germinal center B-cells, long-lived plasma cells, long-term IgG responses, and induction of memory antibody responses. IgG anti-SRBC reduced the amount of SRBC in the spleens of wild-type, but not of FcγR-deficient mice. However, no correlation between suppression and the amount of SRBC in the spleen was observed, suggesting that increased clearance does not explain IgG-mediated suppression. Instead, we found compelling evidence for epitope masking because IgG anti-NP administered with NP-SRBC suppressed the IgG anti-NP, but not the IgG anti-SRBC response. Vice versa, IgG anti-SRBC administered with NP-SRBC, suppressed only the IgG anti-SRBC response. In conclusion, passively transferred IgG suppressed all measured parameters of an antigen-specific antibody/B cell response and an important mechanism of action is likely to be epitope masking.

## Introduction

When antibodies are passively administered together with their specific antigen, they either down- or upregulate the antibody response against this antigen (1–3). IgM, IgG, and IgE enhance antibody responses and their effects depend on interactions of the immune complexes with Fc- or complement receptors, causing more efficient delivery of antigen to splenic B cell follicles and/or causing enhanced presentation of antigen to CD4^+^ T cells. Interestingly, IgG has a dual role and the same monoclonal IgG anti-2,4,6-trinitrophenyl that enhances carrier responses when administered with haptenated proteins, suppresses carrier responses when administered with haptenated erythrocytes (4, 5). Since the 1960s, the suppressive capacity of IgG has been used clinically to protect rhesus D antigen (RhD)-negative women from becoming immunized against fetal RhD-positive erythrocytes entering the maternal circulation through transplacental hemorrhage (6). Administration of IgG anti-RhD to these women has significantly reduced the frequency of hemolytic disease of the fetus and newborn (7). The mechanism behind the dramatic ability of IgG to suppress antibody responses has been elusive. It is of substantial theoretical interest to understand how small amounts of IgG can completely prevent antibody responses to the antigen they recognize. Understanding these mechanisms may also aid in finding effective monoclonal IgG anti-RhD antibodies to use in RhD-prophylaxis.

In the majority of previous studies of IgG-mediated suppression, the number of B cells producing sheep red blood cell (SRBC)-specific IgM during the first week after immunization was analyzed in mice immunized with native SRBC ± IgG anti-SRBC or haptenated SRBC ± IgG anti-hapten. Frequently, more than 99% of early IgM responses were suppressed (8–13). IgG suppresses also primary IgG responses (14–16) and, when administered immediately prior to a secondary immunization, induction of a secondary antibody response (11). Whether IgG can suppress priming for a memory antibody response against SRBC is less clear. Some studies demonstrate that IgG-mediated suppression of priming does occur, although it is usually less efficient than suppression of a primary response (10, 14, 17, 18), while others find no suppression (19). IgG does not prevent priming of T helper cells (10, 15, 16).

The mechanism underlying IgG-mediated suppression has been intensely investigated over the years. Remarkably, no knockout mouse strain has been found in which suppression does not work. It operates well in mice lacking the activating Fc gamma receptors FcγRI, FcγRIII, and FcγRIV (FcRγ KO), the inhibitory FcγRIIB (FcγRIIB KO), the neonatal Fc receptor (FcRn) (β_2_-microglobulin KO) (10, 11, 20, 21), as well as complement factor C1q (C1q KO), complement factor C3 (C3 KO), or complement receptors 1 and 2 (CR1/2 KO) (21). Moreover, a monoclonal IgG1 antibody, which is unable to activate complement, suppresses to the same degree as a complement-activating IgG1 antibody (22). Therefore, it seems unlikely that IgG-mediated suppression is caused by complement-mediated lysis of SRBC. Because suppression is normal in FcγRIIB KO mice, it is also unlikely that IgG suppresses through central B-cell inhibition, where co-crosslinking of the negatively regulating immunoreceptor tyrosine-based inhibitory motif-containing FcγRIIB and the B cell receptor (BCR) induces inhibition of B cell signaling (23). Other mechanisms that could explain IgG-mediated suppression are that IgG increases clearance, without involving complement or Fc-receptors, or that IgG masks epitopes on the antigen, and prevents B cells from binding and becoming activated. Clearance and epitope masking are not mutually exclusive, and mathematical modeling has suggested that both are operative and can act synergistically (24). Passive administration of IgM of different affinities interferes with the development of germinal centers (GCs) and antibody production (25), but whether IgG regulates GC B cells has to our knowledge not been reported previously.

Here, the contribution of clearance and epitope masking has been studied *in vivo* using passively administered polyclonal SRBC- or 4-hydroxy-3-nitrophenyl acetyl (NP)-specific IgG antibodies as suppressors. The data strongly suggest that epitope masking plays a major role for suppression of IgG responses because only responses against the epitopes recognized by IgG were suppressed. No correlation was observed between IgG-mediated reduction of antigen in the spleen and suppression of the antibody response. In addition, we demonstrate for the first time that IgG suppresses the development of specific extrafollicular antibody-secreting cells, GC B cells as well as long-lived plasma cells. Finally, when primary antibody responses were suppressed to a very high degree (>96%), induction of immunological memory was suppressed to the same extent.

## Materials and Methods

### Mice

C57BL/6JBomTac mice (C57BL/6) were from Taconic Bioscience, Inc. (Hudson, NY, USA) and BALB/c mice from Bommice (Ry, Denmark). Fc receptor gamma chain (FcRγ) KO founders were a gift from Ravetch et al. (26) and were backcrossed to BALB/c for 10 generations. FcRγ KO mice lack the common FcRγ-chain and thereby all activating FcγRs associated with this chain (FcγRI, FcγRIII, and FcγRIV). Mice were age and sex matched within each experiment and were bred and maintained in the animal facilities of the National Veterinary Institute (Uppsala, Sweden). This study was carried out in accordance with the recommendations of the Uppsala Animal Research Ethics Committee and the protocol was approved by this committee.

### Antibodies and Antigens Used for Immunizations

Polyclonal IgG^a^ anti-SRBC and polyclonal IgG^a^ anti-NP were prepared from hyperimmune BALB/c serum and polyclonal IgG^b^ anti-SRBC from hyperimmune C57BL/6 serum. IgG was purified by affinity chromatography over a Protein-A Sepharose column (Amersham Pharmacia Biotech, Uppsala, Sweden) (27), dialyzed against PBS, sterile filtered and stored at −20°C until use. IgG^a^ anti-NP was biotinylated using 0.18 mg EZ-Link Sulfo-NHS-LC-LC-Biotin (sulfosuccinimidyl-6-[biotinamido]-6-hexanamido hexaonate) (Thermo Scientific, Waltham, MA, USA) per 2 mg IgG according to manufacturer’s recommendations. The reaction was performed at room temperature for 30 min and free biotin was removed by dialysis against PBS. IgG^a^ anti-NP-biotin was sterile filtered and stored at 4°C until use. SRBC were acquired from Håtunalab AB (Håtunaholm, Sweden) and stored in sterile Alsever’s solution at 4°C. SRBC were washed three times in PBS before use. 4-hydroxy-3-nitrophenylacetic-e-aminocaproyl-OSu (NP-ε-Aminocaproyl-OSu) (Biosearch Technologies, Petaluma, CA, USA) was conjugated to SRBC as described before (16). Briefly, NP-ε-Aminocaproyl-OSu was dissolved in dimethylformamide at a concentration of 7.5 mg/ml. Dissolved NP-ε-Aminocaproyl-OSu were then added to 5% SRBC suspensions in conjugation buffer (0.1 M NaHCO_3_ with 0.15 M NaCl, pH 8.5) to a final concentration of 250 μg/ml (in Figure 3, a final concentration of 1 mg/ml NP-ε-Aminocaproyl-OSu was used in order to achieve higher coupling ratio and facilitate visualization in sections) and incubated for 1 h at room temperature. After the conjugation reaction, cells were washed three times in PBS before use.

### Immunization and Blood Sampling

All mice were immunized with SRBC or NP-SRBC in one of their lateral tail veins in 200 μl PBS. Antigen-specific IgG was always administered in 200 μl PBS 30 min prior to antigen, also *via* the lateral tail veins. Controls received antigen alone or antigen-specific IgG alone. Secondary immunizations were done with SRBC alone. Further details of doses are given in the figure legends. The “default” doses were 30–50 μg IgG and 5 × 10^7^ (NP-)SRBC, both of which are known cause 90–99% suppression of antibody responses to this amount of erythrocytes. In studies of NP-specific antibody-secreting cells (Figure 3), the higher dose 5 × 10^8^ NP-SRBC had to be used to induce detectable numbers of NP-specific cells and the dose of IgG was correspondingly increased to 100 μg. In immunological memory experiments, the lower priming dose 5 × 10^6^ SRBC together with 10 μg IgG was used (Figure 5A) in addition to the default doses (Figure 5B) to test whether the strength of priming affected whether IgG was able to suppress memory induction or not. Blood was collected from the ventral tail artery.

### Enzyme-Linked Immunospot (ELISPOT)

Enzyme-linked immunospot assay was used for measuring SRBC-specific IgG-secreting cells (28). Briefly, 96-well plates (Costar 96-well enzyme immunoassay/RIA; Sigma-Aldrich, St. Louis, MO, USA) were coated with 0.25% SRBC. Spleen cells were diluted in cell culture medium (DMEM with 0.5% FCS), 100 μl was added to each well, and the plates were incubated at 37°C for 2.5 h. SRBC-specific antibodies produced by the single cells were detected after addition of goat anti-mouse IgG-alkaline phosphatase (Jackson ImmunoResearch Laboratories, Inc., West Baltimore Pike, Media, PA, USA) for 3 h at room temperature, followed by addition of the precipitating substrate 5-bromo-4-chloro-3-indolyl phosphate (Sigma-Aldrich) for 30 min at room temperature. The plates were washed three times in PBS and samples were counted blindly under a stereomicroscope.

### Enzyme-Linked Immunosorbent Assay (ELISA)

Also NP- and SRBC-specific antibodies in sera were measured by ELISA (16). Briefly, 96-well plates (Costar 96-well enzyme immunoassay/RIA; Sigma-Aldrich) were coated with either 100 μl 0.25% SRBC or 100 μl NP_20_-BSA (Biosearch Technologies) (50 μg/ml) in PBS with 0.05% NaN_3_. The plates were then blocked with 5% dry milk at 4°C overnight. After washing three times with PBS, serum samples were serially diluted, added to the plates, and incubated overnight at 4°C. When measuring secondary IgG responses, starting serum dilution for titer determination was 1:5. Cutoff for titers was set as mean OD_405nm_ + 2× SD for a group of eight individual sera from naive BALB/c mice diluted 1:5 (29). When measuring the IgG response, depending on the allotype of injected antibodies, either biotinylated anti-IgG1^b^ (clone B68-2) and anti-IgG2a^b^ (clone 5.7) or biotinylated anti-IgG1^a^ (clone 10.9) and anti-IgG2a^a^ (clone 8.3) (all from BD Pharmingen, San Jose, CA, USA) were mixed 1:1, added to each well, and incubated overnight at 4°C. After washing, alkaline phosphatase-conjugated streptavidin (BD Pharmingen, San Jose, CA, USA) was added and incubated for 3 h at room temperature. After washing, plates were developed using the substrate (*p*-nitrophenylphosphate; Sigma-Aldrich). Absorbance at 405 nm was measured and data analyzed using SoftMax software (Molecular Devices, Sunnyvale, CA, USA).

### Flow Cytometry

Spleen cells were prepared as described before (30) and re-suspended in FACS buffer (PBS with 2% fetal bovine serum). Samples were treated with Fc block (anti-CD16/32; BD Biosciences, San Jose, CA, USA) for 10 min on ice, then stained with anti-B220-Alexa700 (clone RA3-6B2), anti-GL7-BV421 (clone GL7), anti-CD95-PEcy7 (clone Jo2), anti-λ1-biotin (clone R11-153) (all from BD Biosciences) at 4°C for 30 min. After washing twice in FACS buffer, samples were stained with streptavidin-FITC and NP-PE at 4°C for 30 min and re-suspended in 300 μl FACS buffer after washing in FACS buffer. For each sample, 2–3 million events were acquired on a LSR Fortessa cytometer (BD Biosciences) at the BioVis platform, SciLifeLab, Uppsala, Sweden. Data were analyzed with FlowJo software (Tree Star Inc., Ashland, OR, USA).

### Confocal Laser Scanning Microscopy

Spleens were harvested, embedded in optimal cutting temperature embedding compound (VWR international, Radnor, PA, USA), flash-frozen in liquid nitrogen, and stored at −80°C. Eight-micrometer sections were cut using Thermo Scientific CryoStar NX70 Cryostat (Thermo Scientific, Waltham, MA, USA), thaw-mounted on frost plus microscope slides (Menzel-Gläser, Braunschweig, Germany), air-dried and stored at −80°C until use. Prior to staining, slides were fixed in 4% paraformaldehyde (Merck, Darmstadt, Germany) in PBS (pH 7.8) for 15 min or in 50% acetone for 30 s followed by 100% acetone (Sigma-Aldrich) for 5 min. Slides were then rehydrated in PBS and blocked with 5% horse serum (Sigma-Aldrich) in PBS for 30 min. Slides were stained with primary antibodies for 1 h. After washing twice in PBS, fluorochrome-conjugated streptavidin was added and the slides were incubated for 1 h and washed twice in PBS prior to mounting with Fluoromount G (Southern Biotech, Birmingham, AL, USA). For detection of NP-specific B cells, slides were stained with anti-IgD-Alexa 488 (clone 11-26C.2a, BioLegend, San Diego, CA, USA) and NP-PE (Biosearch Technologies). Localization of SRBC to splenic marginal zones (MZs) were investigated using anti-B220-Pacific blue (clone RA3-6B2, BD Biosciences), anti-CD169 (MOMA)-FITC (clone MOMA-1, AbD Serotec, Raleigh, NC, USA), and NP-SRBC detection by an in-house produced polyclonal IgG anti-NP-biotin followed by streptavidin-PE (eBioscience, San Diego, CA, USA). Images of immunofluorescence were acquired blindly and randomized with a LSM 700 confocal microscope (Carl Zeiss, Thornwood, NY, USA) using Zen 2009 software (Carl Zeiss). Tile-scan images of whole spleen sections were acquired using Zen 2009 software. Images were analyzed blindly with ImageJ software (NIH, Bethesda, MD, USA).

### Statistical Analysis

Statistical differences between groups were determined by the two-tailed Student’s *t*-test. Statistical significance levels were set as: ns, *p* > 0.05; *p < 0.05; **p < 0.01; ***p < 0.001.

## Results

### IgG-Mediated Decrease of the Amount of SRBC in the Spleen Is Dependent on Activating FcγRs and Does Not Correlate with Suppression

**Figure 1:**
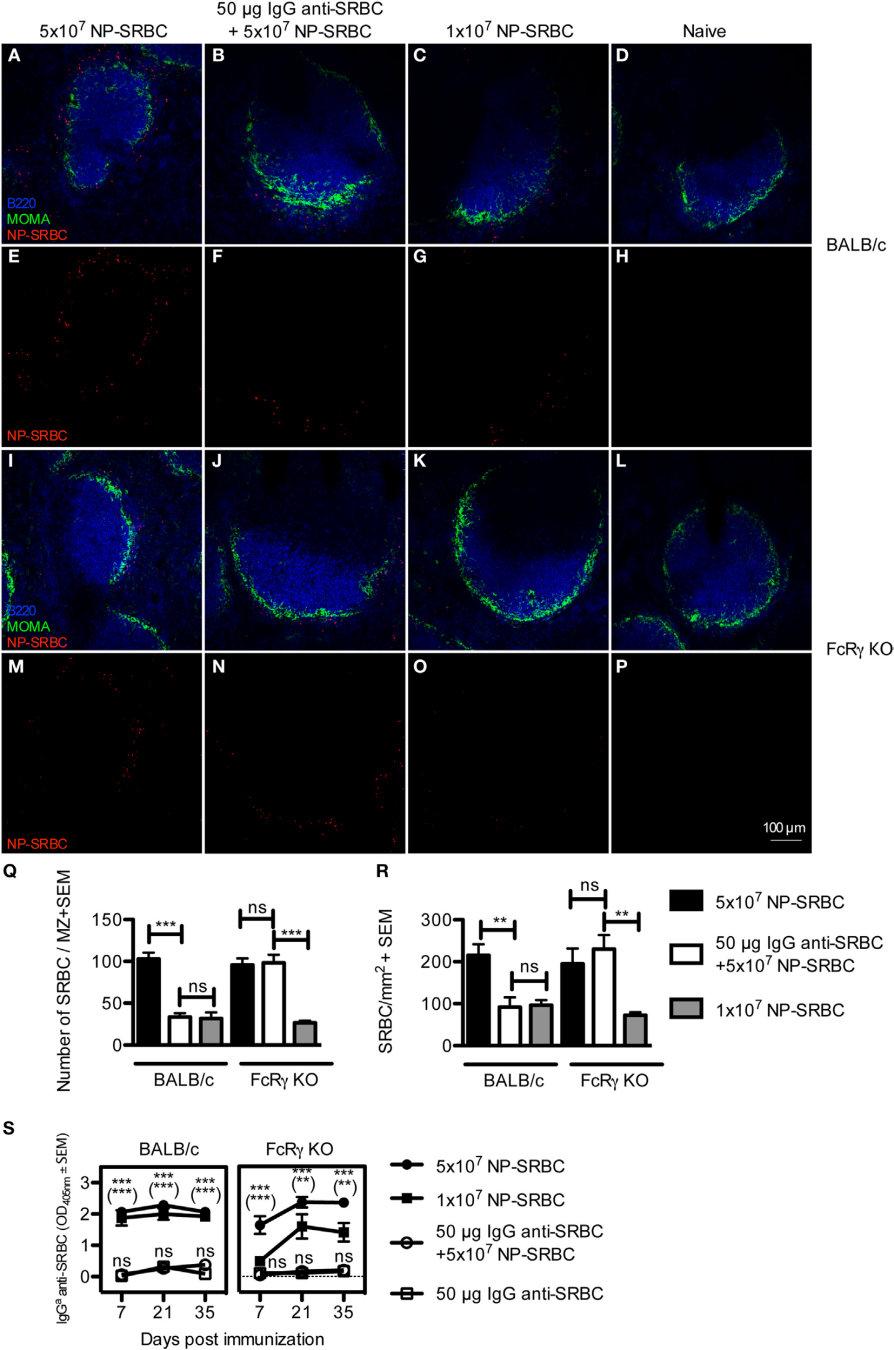
**IgG-mediated decrease of the amount of sheep red blood cell (SRBC) in the spleen is dependent on activating FcγRs and does not correlate with suppression**. BALB/c and FcRγ KO mice were immunized with 5 × 10^7^ NP-SRBC ± 50 μg IgG^b^ anti-SRBC or 1 × 10^7^ NP-SRBC. Naive mice and mice-receiving IgG^b^ anti-SRBC alone were used as negative controls. Spleens were harvested 10 min after administration of NP-SRBC and processed for analysis by confocal laser scanning microscopy (*n* = 5/group). **(A–P)** Visualization of NP-SRBC localization in spleen sections: B220^+^ B cells (blue), MOMA^+^ metallophilic macrophages (green), and NP-SRBC (red). Imaging of samples was performed blindly. **(Q)** Quantification of the number of NP-SRBC per marginal zone. Ten to fifteen randomly selected follicles per spleen per mouse were imaged at 20× magnification. **(R)** Quantification of the number of NP-SRBC per millimeter square in whole spleen sections. Tile scans of one whole spleen section per mouse were imaged at 10× magnification. **(S)** The IgG anti-SRBC response was followed in parallel in mice from each group for 7–35 days after immunization (*n* = 5/group, *n* = 3 for negative controls). Sera diluted 1:125 were screened for IgG^a^ anti-SRBC in enzyme-linked immunosorbent assay. *p*-Values for comparisons of mice immunized with IgG anti-SRBC and NP-SRBC vs 5 × 10^7^ NP-SRBC alone are given without parentheses. Comparisons of mice immunized with IgG anti-SRBC and 5 × 10^7^ NP-SRBC vs 1 × 10^7^ NP-SRBC alone are given within parentheses. Data are representative of two (S: BALB/c), one (S: FcRγ KO), or at least three experiments **(A–R)**. ns, *p* > 0.05; *p < 0.05; **p < 0.01; ***p < 0.001.

Specific IgG administered together with SRBC reduces the localization of SRBC in the MZ of the spleen and increases clearance of SRBC from the blood (16). However, after 10 min, the levels of SRBC in the circulation were undetectable, regardless of whether IgG had been co-administered or not (16). The correlation between suppression and antigen localization was not directly assessed nor was the importance of FcγRs studied. To investigate this, BALB/c and FcRγ KO mice were immunized with IgG anti-SRBC + 5 × 10^7^ NP-SRBC, 5 × 10^7^ NP-SRBC alone, or with 1 × 10^7^ NP-SRBC alone. Spleens were harvested after 10 min and the amount of extracellular NP-SRBC was determined by confocal laser scanning microscopy (Figures 1A–R; enhanced images are shown in Figure S1 in Supplementary Material). In parallel, the serum IgG anti-SRBC response was followed in groups of mice immunized at the same time (Figure 1S). Because the passively administered IgG was obtained from mice with a different IgG allotype than the recipient mice, the actively produced endogenous IgG antibodies could be distinguished by an allotype-specific ELISA (21). Administration of IgG significantly reduced the amount of SRBC in the MZ of wild-type BALB/c mice (Figures 1B,F vs. Figures 1A,E; Figure 1Q) as well as in the entire spleen section (Figure 1R; Figure S2 in Supplementary Material). BALB/c mice immunized with 1 × 10^7^ NP-SRBC alone, or with the fivefold higher amount of NP-SRBC together with IgG anti-SRBC, had comparable amounts of NP-SRBC in their spleens (Figures 1C,G,R). In spite of this, mice immunized with 1 × 10^7^ NP-SRBC alone mounted a potent antibody response while mice immunized with IgG anti-SRBC + 5 × 10^7^ NP-SRBC had a suppressed antibody response (Figure 1S). Some spleens were also analyzed for NP-SRBC after 1 h and 24 h, but, as expected, very little or no antigen could be detected at these time points [Ref. (31) and data not shown]. In FcRγ KO mice, IgG was unable reduce the amount of SRBC in the MZ (Figures 1J,N,Q) and in the entire spleen (Figure 1R). In spite of the similar amounts of SRBC in the spleens, the antibody response was suppressed in the IgG group (Figure 1S).

In summary, in BALB/c mice, equal (low) levels of antigen in the spleen can result in high (1 × 10^7^ NP-SRBC-group) or suppressed (IgG + 5 × 10^7^ NP-SRBC-group) antibody responses. In FcγR KO mice, equal (high) levels of antigen can result in high (5 × 10^7^ NP-SRBC) or suppressed (IgG + 5 × 10^7^ NP-SRBC-group) antibody responses. This lack of correlation between suppression and the amount of NP-SRBC detected in the spleen is hard to reconcile with clearance of antigen as the major explanation for IgG-mediated suppression. Moreover, the observations confirm that administration of SRBC-specific IgG reduces the amount of antigen localized in the spleen (16) and demonstrate that the reduction is dependent on activating FcγRs while the suppression of antibody responses is not.

### Epitope-Specific Suppression of IgG Responses

**Figure 2:**
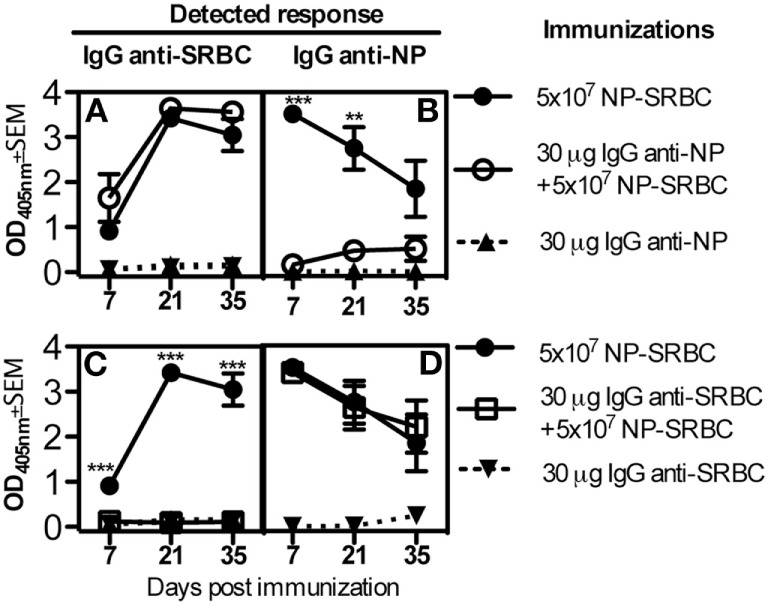
**Epitope-specific suppression of IgG responses**. C57BL/6 mice were immunized with 5 × 10^7^ NP-sheep red blood cells (SRBC) ± 30 μg IgG^a^ anti-NP or **(A,B)** with 5 × 10^7^ NP-SRBC ± 30 μg IgG^a^ anti-SRBC **(C,D)**. IgG was also administered alone as negative control. Serum samples were diluted 1:125 and analyzed in enzyme-linked immunosorbent assay. Representative of four **(A,B)** or two **(C,D)** experiments; *n* = 5 mice/group (except for IgG alone where *n* = 2 mice/group). Statistical comparisons were done between solid circles vs. open circles in **(A,B)** and between solid circles vs. open squares in **(C,D)**. ns, *p* > 0.05; *p < 0.05; **p < 0.01; ***p < 0.001.

Next, we sought to determine whether IgG only suppresses responses against the epitopes to which it binds (epitope-specific suppression) or whether it, in addition, suppresses responses against other epitopes on the same antigen (non-epitope specific suppression). To this end, C57BL/6 mice were immunized with NP-SRBC alone or together with either IgG anti-NP or IgG anti-SRBC. The mice were bled every 2 weeks and their antibody responses against NP and SRBC were analyzed. IgG anti-NP suppressed the NP- but not the SRBC-specific IgG-responses (Figures 2A,B) while IgG anti-SRBC suppressed the SRBC- but not the NP-specific IgG response (Figures 2C,D). These observations were highly reproducible (4/4 experiments with IgG anti-NP and 2/2 with IgG anti-SRBC) and demonstrate that IgG is able to suppress IgG responses in an epitope-specific way.

### IgG Suppresses NP-Specific Extrafollicular Antibody-Secreting Cells and NP-Specific GC B Cells

**Figure 3:**
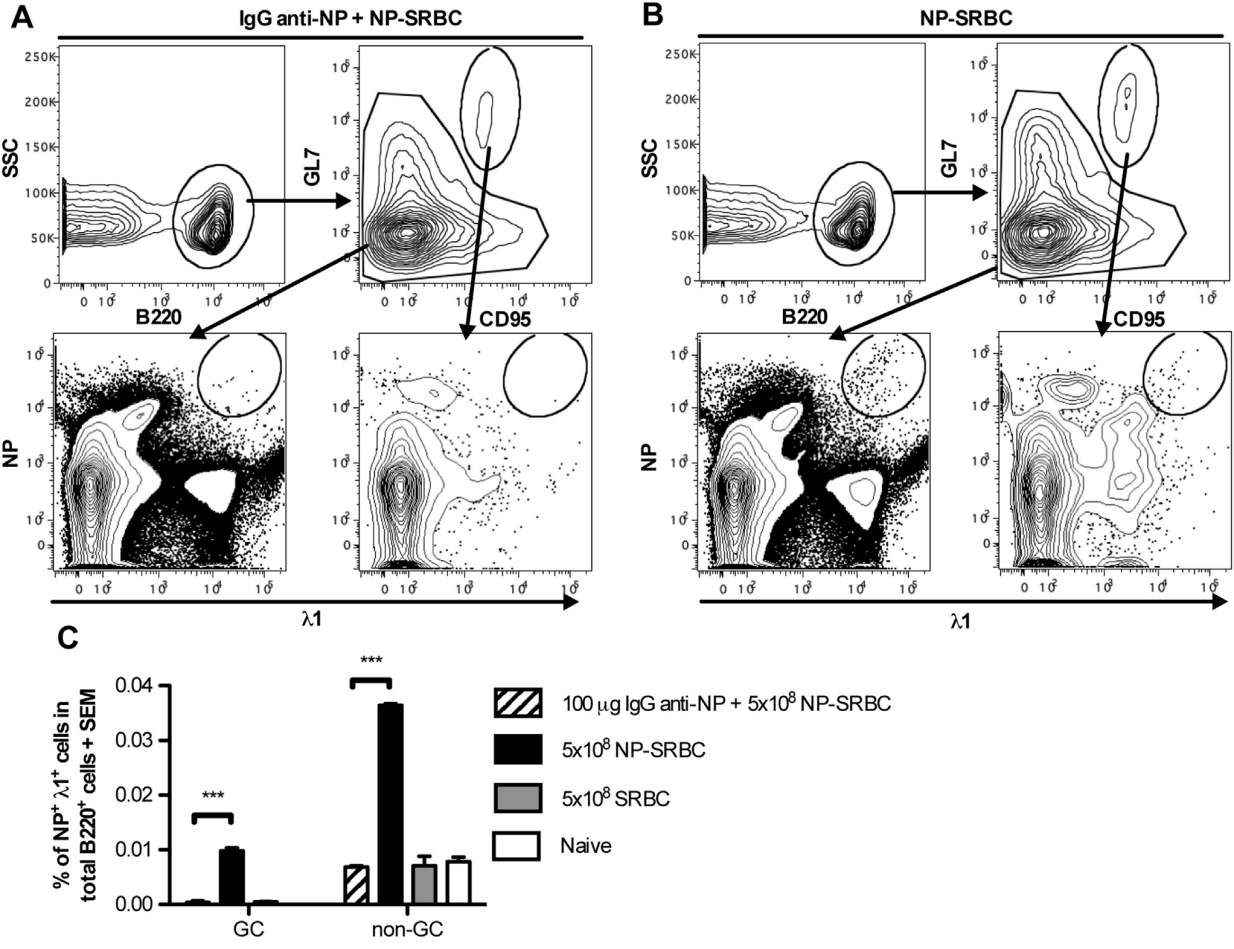

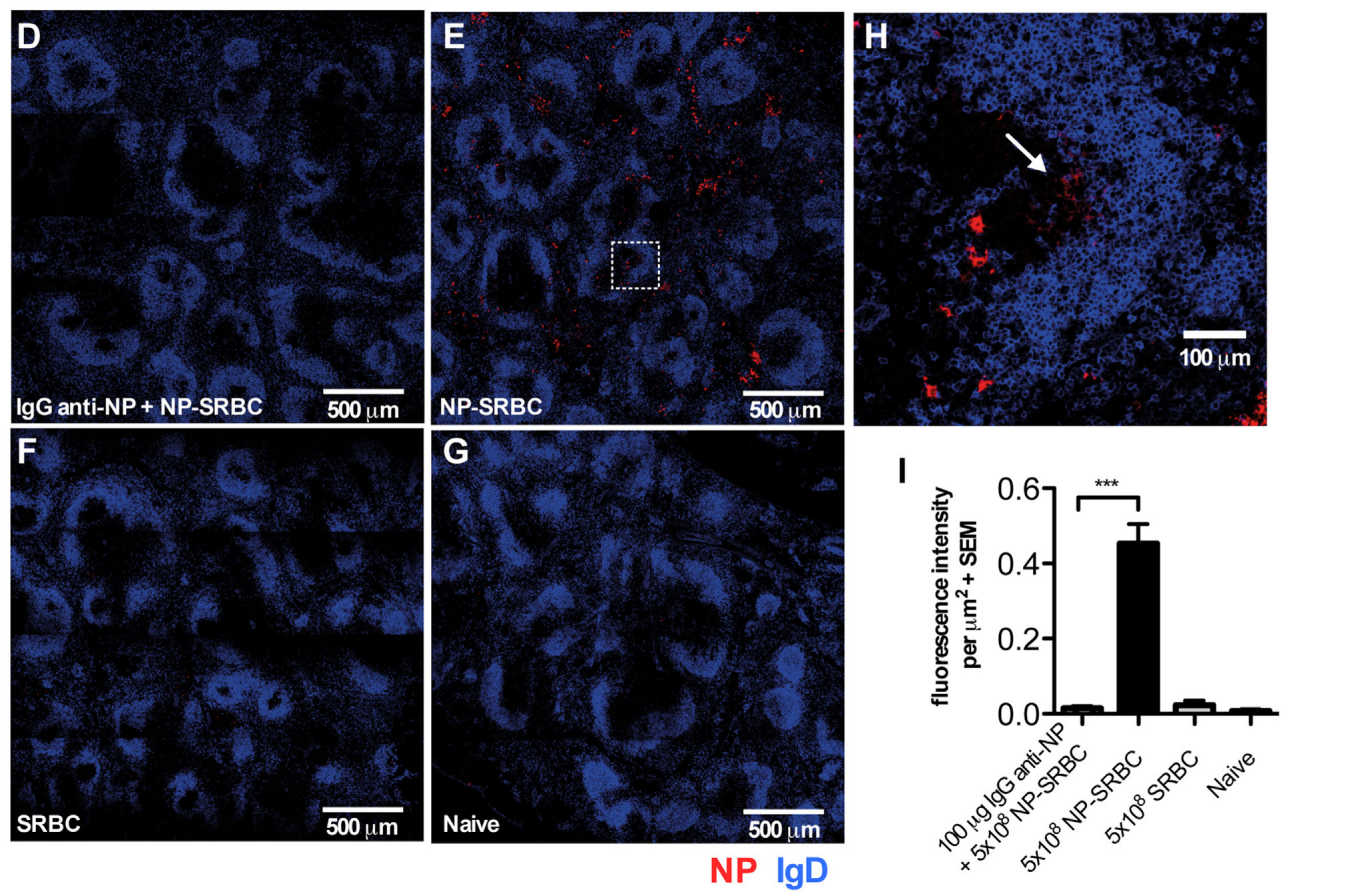
**IgG suppresses NP-specific extrafollicular antibody secreting cells and NP-specific germinal centers (GCs) B cells**. C57BL/6 mice were immunized with 5 × 10^8^ NP-sheep red blood cell (SRBC) ± 100 μg polyclonal IgG^a^ anti-NP, 5 × 10^8^ unconjugated SRBC, or left untreated. After 6 days, spleens were harvested and half of the spleen samples were analyzed with flow cytometry **(A–C)** and the other half with confocal microscopy **(D–I)**. **(A,B)** Gating strategy for GC and non-GC NP^+^λ1^+^ cells. **(C)** Frequency of GC and non-GC NP^+^λ1^+^ cells among total B220^+^ cells. **(D–G)** Representative tile scan sections, stained for IgD^+^ (blue), and NP^+^ (red) cells. **(H)** Enlarged image from **(E)** (square with dashed lines). **(I)** Quantification of fluorescence intensity per square micrometer. One representative tile scan image was taken from each mouse and the intensity of red fluorescence (NP^+^) from each image was summed and then divided by the size of the image. Representative of three independent experiments with 2–4 mice/group. ns, *p* > 0.05; *p < 0.05; **p < 0.01; ***p < 0.001.

In previous studies, the ability of IgG to suppress the overall serum antibody levels or splenic antibody-secreting cells was analyzed. Here, we investigated which B cell subpopulations were suppressed by IgG, using a system in which the BCRs of the antigen-specific B cells could be stained directly. The antibody response against NP in C57BL/6 mice is genetically restricted, mainly comprising λ1 light chains and the V186.2 segment of the VHJ558 gene family (32). Therefore, staining for λ1 and NP can be used to identify NP-specific B cells. C57BL/6 mice were immunized with NP-SRBC ± IgG anti-NP and negative controls with unconjugated SRBC or left unimmunized. Six days later, spleens were analyzed for NP-binding cells with flow cytometry and confocal microscopy (Figure 3). In mice immunized with NP-SRBC alone, a small population of λ1^+^NP^+^B220^+^ cells were identified both among the GL7^high^ CD95^high^ GC B cells (Figure 3B bottom right panel; Figure 3C) and among the GL7^low^CD95^low^ non-GC B cells (Figure 3B bottom left panel; Figure 3C). The NP-specific cells (GC B cells and non-GC B cells together) constituted approximately 0.05% of the total B220^+^ population and ~1/5 were GC B cells and ~4/5 non-GC B cells (Figure 3C). Administration of IgG anti-NP together with NP-SRBC abolished the induction of NP-specific GC and non-GC B cells (Figures 3A,C). In fact, the levels in these mice were equally low as in mice immunized with unconjugated SRBC or left untreated (Figure 3C).

The other half of the spleens were analyzed in confocal microscopy (Figures 3D–I). NP-binding cells were readily detected in extrafollicular foci in mice immunized with NP-SRBC (Figure 3E) while very few NP-binding cells were detected in mice immunized with IgG anti-NP + NP-SRBC (Figure 3D) and in the negative controls (Figures 3F,G). Although it cannot be excluded that some of the NP-binding cells in extrafollicular foci had exited GCs, the majority are presumably true extrafollicular cells owing to the early time point at which they were analyzed. Importantly, all NP-specific cells outside the follicles, regardless of their origin, are absent in mice immunized with IgG anti-NP + NP-SRBC. NP-specific cells could also be detected in some GCs (Figure 3H). GC B cells stained less brightly than the extrafollicular NP-specific cells possibly due to a wider span in affinity (33) and lower Ig expression. To confirm that the unstained areas close to the IgD^+^ areas are indeed GCs, co-staining with PNA was performed (Figure S3 in Supplementary Material). In summary, IgG anti-NP can suppress the generation of both extrafollicular NP-specific antibody-secreting cells and NP-specific GC B cells.

### IgG Suppresses the Generation of Long-Lived Plasma Cells

**Figure 4:**
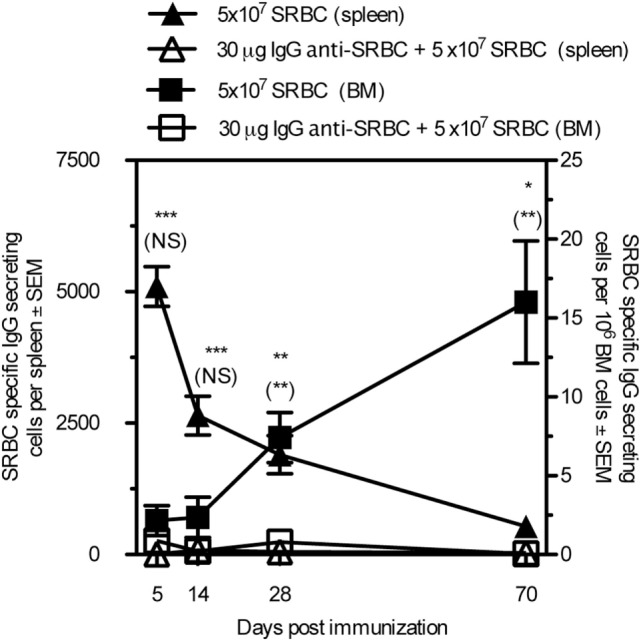
**IgG suppresses the generation of long-lived plasma cells**. BALB/c mice were immunized with 5 × 10^7^ sheep red blood cell (SRBC) ± 30 μg IgG^b^ anti-SRBC. Spleens and bone marrow were harvested and SRBC-specific antibody-secreting cells were measured with ELISPOT. Comparisons between filled and open triangles are given without parentheses and comparisons between filled and open squares are given within parentheses. ns, *p* > 0.05; *p < 0.05; **p < 0.01; ***p < 0.001.

To determine whether IgG suppressed the development of long-lived plasma cells, mice were immunized with SRBC ± IgG anti-SRBC and the number of SRBC-specific IgG-secreting cells in the spleen and bone marrow was analyzed 5–70 days after immunization (Figure 4). Cells secreting IgG anti-SRBC were detected in both organs at all times, but were severely suppressed in the groups receiving IgG together with SRBC. As expected, the number of antibody-producing cells in the spleen was highest early after immunization and then decreased, while the cell number in the bone marrow increased during the entire time period. Thus, administration of IgG anti-SRBC efficiently suppressed the specific IgG-secreting cells at all times, both in spleen and bone marrow.

### IgG Suppresses Induction of Immunological Memory

**Figure 5:**
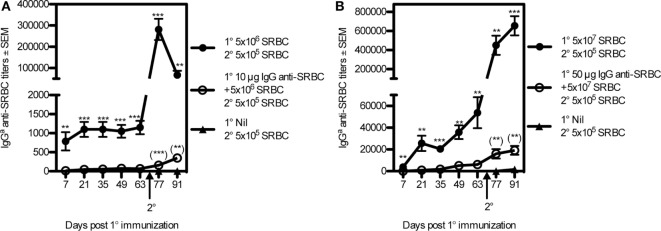
**IgG suppresses the induction of immunological memory**. BALB/c mice were immunized with 5 × 10^6^ sheep red blood cell (SRBC) ± 10 μg IgG^b^ anti-SRBC+ or **(A)** or 5 × 10^7^ SRBC ± 50 μg IgG^b^ anti-SRBC **(B)**. And, 70 days after priming, all mice were boosted with 5 × 10^5^ sheep red blood cells (SRBCs). An additional, unprimed, group also received 5 × 10^5^ SRBC at this time point (filled triangles). Serum IgG^a^ anti-SRBC titers were assayed in enzyme-linked immunosorbent assay. *p*-Values for comparisons between mice primed with IgG anti-SRBC and 5 × 10^7^ SRBC vs. 5 × 10^7^ SRBC alone are given without parentheses. Comparisons between mice primed with IgG anti-SRBC and SRBC and boosted with 5 × 10^5^ SRBC vs. naive mice “boosted” with 5 × 10^5^ SRBC alone are given within parentheses. Data are representative of at least three experiments per antigen dose (*n* = 5–8/group). ns, *p* > 0.05; **p < 0.01; ***p < 0.001.

To test the ability of IgG to regulate memory induction, BALB/c mice were immunized in two regimes: 5 × 10^7^ SRBC ± 50 μg IgG anti-SRBC or 5 × 10^6^ SRBC ± 10 μg IgG anti-SRBC. The IgG anti-SRBC responses were followed during 63 days after priming, confirming efficient suppression (Figures 5A,B). And, 70 days after priming, all mice were boosted with a suboptimal number of SRBC and, in addition, a group of naive mice received the same “booster” dose. Because of the great differences in antibody levels between the groups, we analyzed the antibody responses as endpoint titers, starting at a serum dilution of 1:5. In mice that had been primed with SRBC alone and boosted, a very high secondary antibody response was observed (Figures 5A,B). In mice primed with IgG + SRBC and boosted, secondary responses were low (Figures 5A,B). A minute priming effect was, however, visible also in the IgG groups, evidenced by the fact that their antibody titers were higher than those in naive and “boosted” mice (Figures 5A,B). Naive mice, primed on day 70 with the suboptimal dose 5 × 10^5^ SRBC, produce low titers of IgG antibodies [mean titers: 30 (Figure 5A) and 1,100 (Figure 5B)], which are barely visible in the figures. The relative suppression of primary responses and induction of memory appeared to be equal. In the low dose experiment, IgG suppressed 96% of the IgG-response on day 21 and 99% on day 91 (21 days after boost) (Figure 5A). In the high dose experiment, suppression was 97% both 21 days after priming and 21 days after boost (Figure 5B). Thus, when IgG-mediated suppression of primary antibody responses is very efficient, priming for secondary antibody responses seems to follow along the same lines.

## Discussion

Generation of an adaptive antibody response is a complicated process, and one purpose of the present investigation was to define in detail which parameters along this line that can be suppressed by IgG. After the initial interaction between antigen, B cells, and cognate T cells, the specific B cells can differentiate into extrafollicular short-lived IgM- or IgG-secreting cells, or GC-independent memory B cells. Other B cells enter the GC pathway, go through hypermutation followed by selection for high affinity and class switch recombination becoming either GC-dependent memory B cells or long-lived plasma cells [reviewed in Ref. (34–36)]. Differentiation between IgG-mediated suppression of extrafollicular B cells, non-GC B cells, and GC B cells in the spleen is not possible by just looking at serum antibodies or direct plaque-forming cell assay. In order to detect single antigen-specific B cells *in vivo*, usually Ig-transgenic mice immunized with antigen in adjuvants are used. Here, we managed to set up a system in which i.v. immunization of C57BL/6 mice with 5 × 10^8^ NP-SRBC in PBS resulted in NP-specific B cell populations, which were detectable through staining of specific BCR with NP-PE. Administration of IgG anti-NP completely prevented development of NP-specific extrafollicular B cells, non-GC B cells, and GC B cells (Figure 3). IgG anti-SRBC also suppressed the development of long-lived plasma cells in bone marrow obtained up to 70 days after immunization (Figure 4).

An important function of the adaptive immune system is the generation of long-lived memory B cells. Whether IgG, acting to suppress a primary response, also inhibits priming for immunological memory has not been unequivocally determined (10, 14, 17–19). By using an ELISA that discriminates between passively administered and actively produced, endogenous IgG, and by measuring the IgG levels in the serum as endpoint titers, we could directly compare the relative suppression of primary and secondary IgG responses. The data show that when primary responses were suppressed by 96% or more, secondary responses were also suppressed by 96% or more. Thus, the relative suppression of a primary IgG response and priming for a secondary IgG response is very similar (Figure 5). This is in accordance with current knowledge about the development of memory B cells, most of which are generated during the first weeks of an immunization (36). Therefore, it would be expected that lack of B cell stimulation, owing to IgG-mediated inhibition of the interaction between antigen and B cells, during this time, would result in lack of memory B cells. When the primary B cell response is only partially suppressed, a partial suppression of priming would be the logical result, and this may explain why priming has sometimes been found to be only moderately suppressed. The observation is also consistent with the successful RhD prophylaxis in humans in which administration of IgG anti-RhD to women at the first pregnancy with a RhD-positive baby protects against immunization during the next pregnancy (6, 7).

To further elucidate the mechanism underlying IgG-mediated suppression, we focused on differentiating between the two hypotheses that are currently mainly discussed, antigen clearance and epitope masking. Provided FcγRs were expressed in the mice, IgG was able to increase clearance, but no correlation between the antibody response and the amount of NP-SRBC found in the spleens was observed (Figure 1). These findings are consistent with previous observations. IgG administered several days after SRBC causes suppression (11, 12, 37–40) in spite of the fact that SRBC is cleared from the circulation of mice within 10 min of immunization (16). In a model using as antigen transgenic mouse erythrocytes (HOD-RBC), which express hen egg lysozyme in tandem sequence with T cell determinants of ovalbumin (OVA) and the complete human Duffy^b^ transmembrane protein (41), a panel of monoclonal IgG antibodies were tested for ability to suppress the OVA-specific antibody response. Three of the antibodies induced clearance while three did not, but all six were efficient suppressors (42). In addition, monoclonal anti-RhD administered together with RhD^+^ erythrocytes to RhD^−^ subjects induced rapid clearance of erythrocytes but failed to suppress the antibody response (43, 44).

Direct evidence for a role of epitope masking in IgG-mediated suppression is difficult to obtain, and this hypothesis will probably remain a “diagnosis by exclusion.” Epitope masking by IgM antibodies was, however, suggested to play an important role in selection of B cells in germinal centers (25), and to be the most important explanation for poor secondary antibody responses to the stem of the influenza surface hemagglutinin during vaccinations (45). The highly reproducible epitope specificity of suppression of the IgG responses shown in Figure 2 lends strong support for the epitope masking hypothesis. This finding may be the closest to a direct proof for the epitope masking hypothesis, which is possible to obtain. Importantly, epitope specificity of suppression has been reported previously (12, 46). However, also non-epitope specific suppression has been observed, mainly in systems studying IgM-producing single B cells during the first week of immunization (4, 9, 10, 13, 16, 22, 42). Because non-epitope specificity has been interpreted as evidence for Fc-dependence of suppression and, therefore, to argue against the epitope masking mechanism, this is an important issue. The existence of non-epitope specific suppression needs to be accommodated with the increasing support for Fc-independence of suppression obtained from studies of FcγR- and complement-deficient mice (10, 11, 20–22) and the observations that F(ab′)_2_ fragments can suppress (10, 20, 47, 48). We have suggested (10, 49), and still believe, that the best explanation for the apparent paradox of lack of Fc-dependence and existence of non-epitope specific suppression is steric hindrance. IgG binding to an epitope present at high density prevents B cells from binding to the specific epitope (*via* epitope masking) as well as to neighboring non-specific epitopes (*via* steric hindrance). When IgG binds to an epitope present at low density, it would only prevent B cells from binding to the specific epitope. Thus, in conditions with high epitope density, non-epitope specific suppression will be observed and in conditions with low epitope density, epitope-specific suppression will take place. With this reasoning, both epitope-specific and non-epitope-specific suppression can be understood, although suppression is independent of the IgG(Fc) part. Experimental support for this idea comes from studies where hapten-specific IgG antibodies suppressed non-epitope-specific SRBC-responses when administered together with high hapten-density SRBC, but not with low hapten-density SRBC (5, 16). In analogy, the amount of IgG that binds to SRBC correlates with how efficiently suppression is induced (9, 50), mixtures of passively administered different monoclonal IgG antibodies induce more efficient suppression than administration of saturating doses of each IgG antibody alone (9, 51, 52), and high affinity of IgG promotes efficient suppression (13, 53). In humans, a strong argument against the epitope masking mechanism has been a study in which anti-Kell antibodies could suppress responses against RhD epitopes (54). However, it was recently shown that the RhD and Kell antigens are positioned closely together on human erythrocytes (55) and, therefore, these observations may perhaps be explained by epitope masking in combination with steric hindrance.

Compatible with epitope masking is also the lack of correlation between clearance and suppression, discussed above, and the fact that T cells are not suppressed by IgG (10, 15, 16). This is what would be expected because epitope masking would not prevent antigen from being internalized and processed by the APCs, which subsequently activate CD4^+^ T cells. Obviously, some experimental data are not compatible with the epitope masking hypothesis. For example, F(ab′)_2_ fragments have been reported to be unable to suppress (4, 13, 17). This can possibly be explained by increased elimination of these fragments owing to lack of binding to FcRn (56), but this issue needs further experimentation. Another argument against epitope masking comes from the HOD-RBC model, in which a monoclonal IgG anti-Duffy antibody was able to non-epitope specifically suppress anti-HEL responses although it did not sterically block binding of serum IgM anti-HEL to HOD-RBC (42). However, it remains to be elucidated how well competition with serum IgM resembles the *in vivo* situation where B cells themselves compete with the suppressive IgG.

In addition to the use of suppressive IgG in RhD prophylaxis, an important clinical issue is the negative impact of passively transferred pathogen-specific maternal antibodies on vaccination of infants [reviewed in Ref. (57–59)]. Also within this field, the mechanisms discussed are FcγRIIB-mediated inhibition, clearance, and epitope masking, but so far, no concensus has been reached.

In summary, the current study extends our understanding of IgG-mediated suppression in two principal ways. First, it suggests that IgG administered in close temporal relationship to antigen inhibits all pathways of B cell differentiation. Serum IgG as well as extrafollicular B cells, non-GC B cells, GC B cells, long-lived plasma cells, and memory B cells are all suppressed. Second, it provides strong evidence for the importance of epitope masking and suggests that the role of IgG-mediated antigen clearance is negligible.

## Author Contributions

JB and HX: designed and performed experiments, and wrote the manuscript. BH: designed experiments and wrote the manuscript.

## Conflict of Interest Statement

The authors declare that the research was conducted in the absence of any commercial or financial relationships that could be construed as a potential conflict of interest.

## Abbreviations

BCR, B cell receptor; C1q, complement factor 1q; C3, complement factor 3; ELISPOT, enzyme-linked immunospot assay; ELISA, enzyme-linked immunosorbent assay; FcγR, Fc gamma receptor; FcRγ, Fc receptor gamma chain; FcRn, the neonatal Fc receptor; GC, germinal center; HEL, hen egg lysozyme; HOD, HEL-OVA-duffy tandem protein; ITIM, immunoreceptor tyrosine-based inhibitory motif; MZ, marginal zone; NP, 4-hydroxy-3-nitrophenyl acetyl; OVA, ovalbumin; PFC, plaque-forming cell assay; RhD, rhesus D antigen; SRBCs, sheep red blood cells; TNP, 2,4,6-trinitrophenyl.
