# Supplementary material for: Epitope-Specific Suppression of IgG Responses by Passively Administered Specific IgG: Evidence of Epitope Masking
Source: Front Immunol. 2017 Mar 6;8:238. doi: 10.3389/fimmu.2017.00238 (PMC5337509; doi:10.3389/fimmu.2017.00238)
Supplement: Supplementary file 1 [file Image_1.PDF]

Supplementary Material

Epitope specific suppression of IgG responses by passively administered IgG

Joakim J.E. Bergström[1,2], Hui Xu[1,2] and Birgitta Heyman*[1]
1) Department of Medical Biochemistry and Microbiology, Uppsala University, Uppsala, Sweden
2) Shared first authorship
*) Corresponding author: Birgitta Heyman, birgitta.heyman@imbim.uu.se

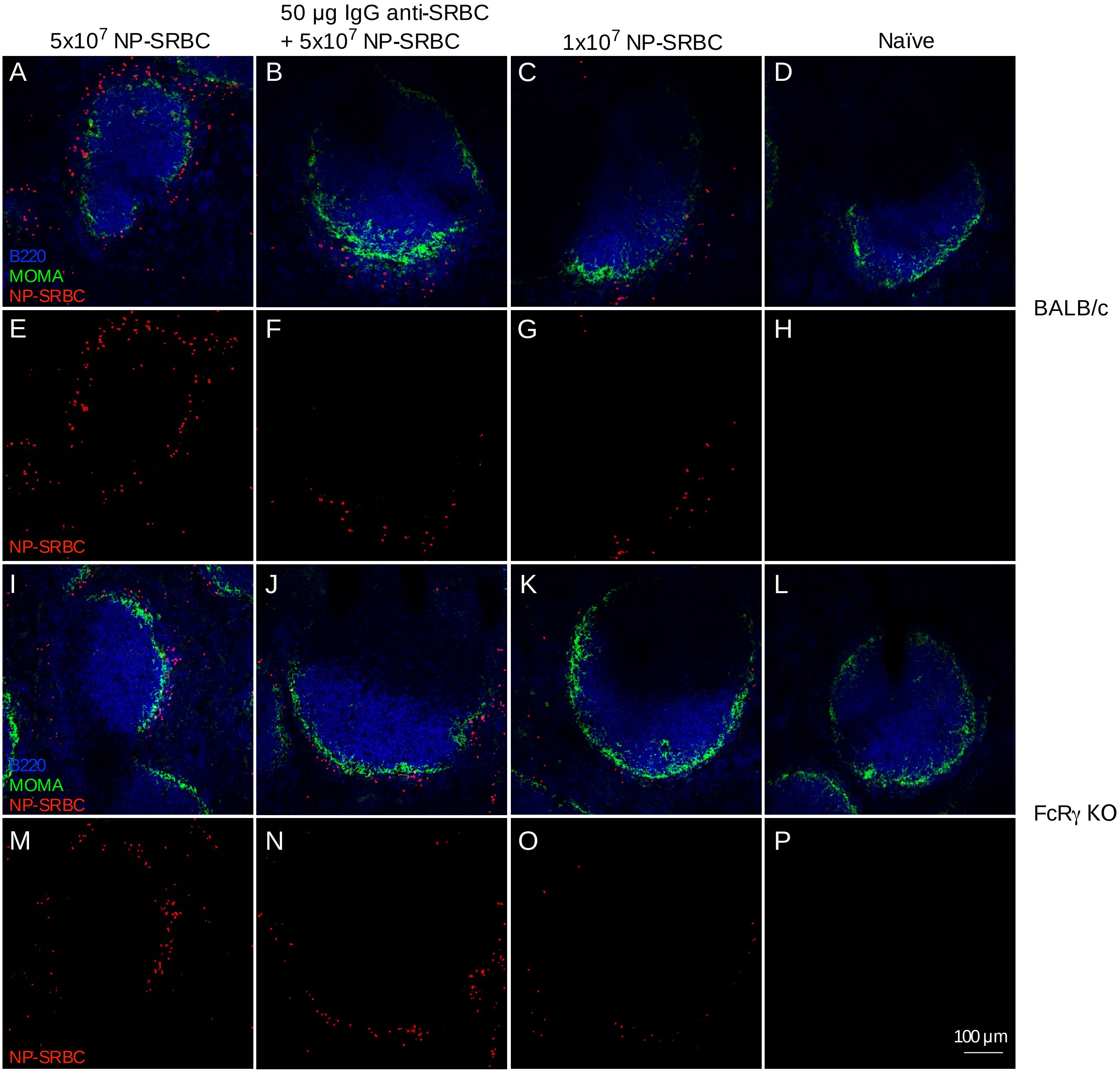

Supplemental Figure 1. IgG-mediated decrease of the amount of SRBC in the spleen does not explain suppression and is dependent on activating FcγR. A-P) Panels are from Figure 1 and NP-SRBC signals above are enhanced using the find edge tool in ImageJ. This was in order to visualize the NP-SRBC particles more clearly. The NP-SRBC signals in the original Figure 1 enclosed in the manuscript is not modified in this manner.

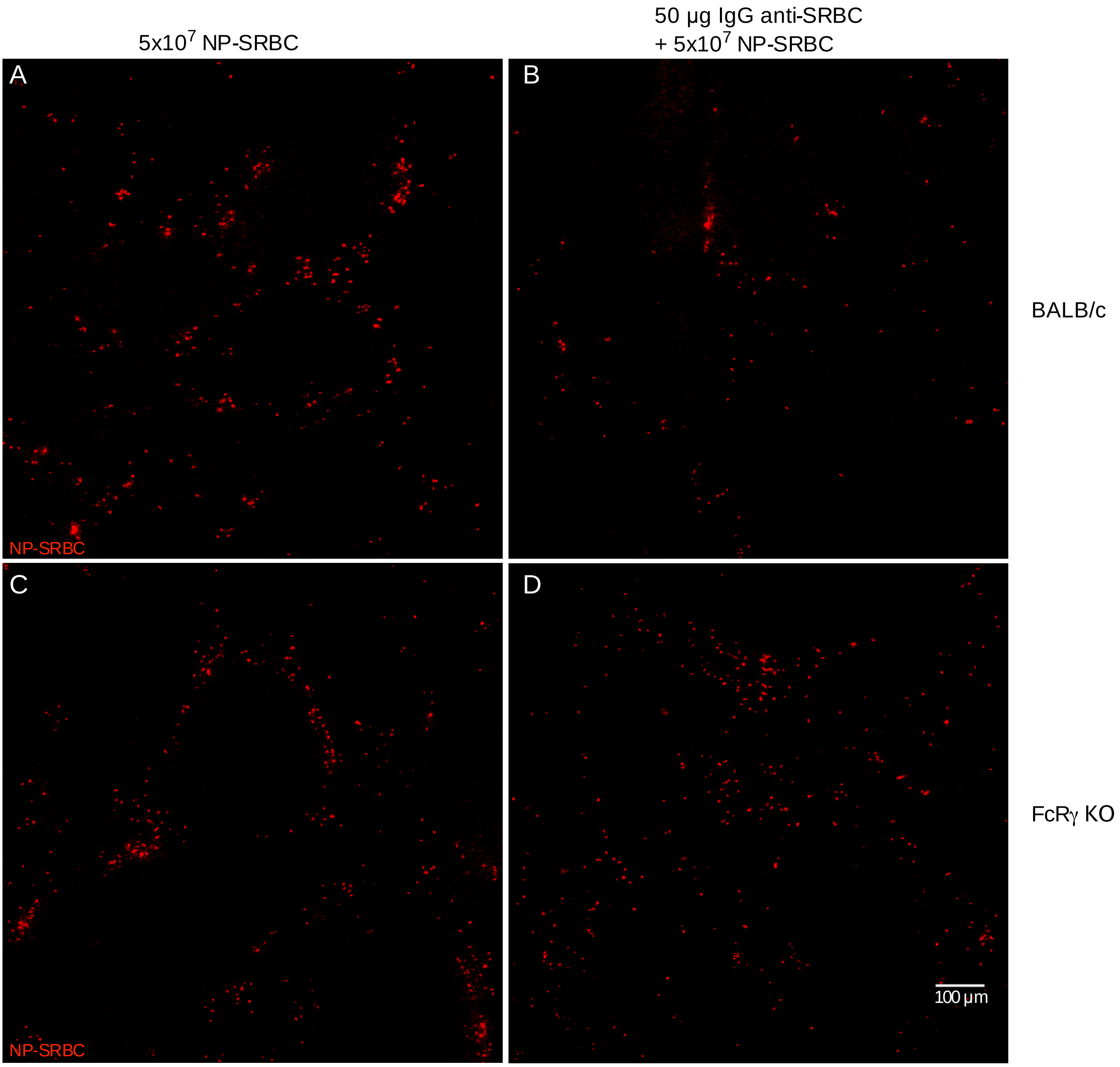

Supplemental Figure 2. IgG decreases the amount of SRBC in the spleens of BALB/c but not FcγR KO mice. A-D) Representative 1 mm^2 areas from tile scans of whole spleen sections from BALB/c (A,B) or FcRγ KO mice (C,D) 10 min after immunization with 50 µg IgG anti-SRBC + $5x10^7$ NP-SRBC (B, D) or $5x10^7$ NP-SRBC alone. Scale bar is 100 µm.

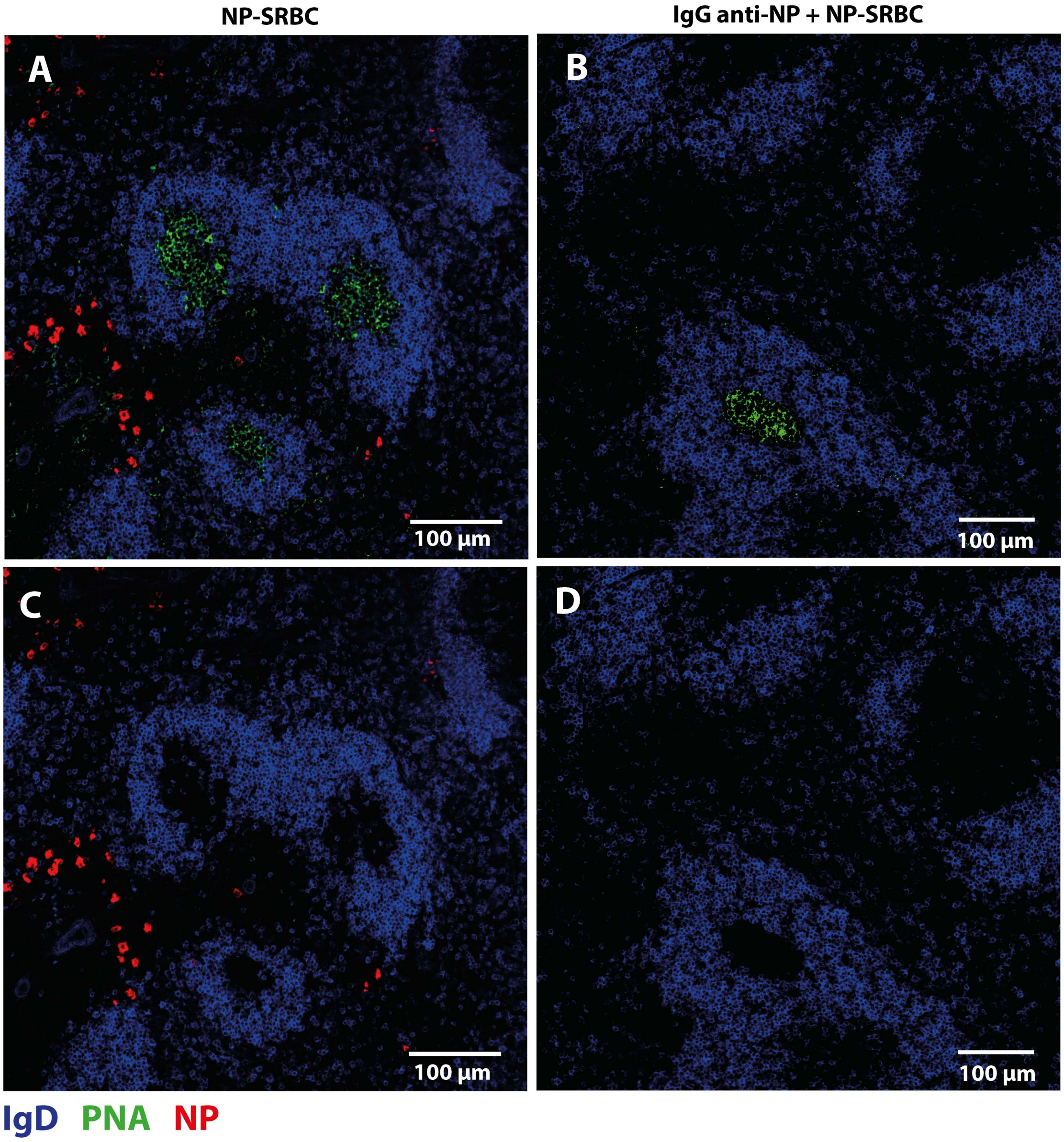

Supplemental Figure 3. IgG suppresses extrafollicular NP-specific B cells A, B) Representative follicles of spleen sections from BALB/c mice 6 days after immunization with $5x10^8$ NP-SRBC (A) or 100 µg IgG anti-NP + $5x10^8$ NP-SRBC (B), stained for IgD^+ (blue), PNA^+ (green), and NP^+ (red) cells. C and D are the same images as A and B with the PNA signal subtracted.
